# Risk assessment and treatment - Evaluation of a group therapy for people with pedophilia

**DOI:** 10.1192/j.eurpsy.2024.775

**Published:** 2024-08-27

**Authors:** P. Heindl, S. Schobel, K. Fischer, T. Nenov-Matt, A. Chrobok, M. Wertz, K. Schiltz

**Affiliations:** Forensic Department, LMU Kinikum, Munich, Germany

## Abstract

**Introduction:**

Deviant sexual interest for children (pedophilia, hebephilia) is associated with a higher risk of sexual offending against children (CSA) and consuming child sexual abuse images (CSAI). There is a general shortage of therapeutic programs for individuals who feel sexually attracted to juvenile bodies and are concerned about their sexual behaviour. Efforts to establish regional centres throughout Germany offering preventive support led to the prevention network “Don’t become an offender” (“Kein Täter werden”).

**Objectives:**

To identify dynamic risk factors (DRFs) and evaluate a treatment programme aiming to reduce CSA and CSAI among potential or existing pedosexual offenders (who have not been legally charged). In addition, changes in the course of therapy are examined to provide information about the accessibility and motivation of the target group and its therapeutic responsiveness.

**Methods:**

Participants undergo standardized diagnostic and treatment procedures. Therapy comprises an outpatient psychotherapy program (group therapy) over the course of approx. 48 weekly sessions, optional individual and partner/relative including sessions, as well as additional pharmaceutical treatment. Assessments are carried out through self- and other-reported psychometric test batteries pre-, during and post-treatment up to a 3.5 year follow-up. The test battery includes clinical questionnaires (WHO-5, CTQ-SF), personality questionnaires (ISK-K, NEO-FFI), sexuality questionnaires (EKK-R, KV-M, MSI, HBI-19) and risk assessment procedures (VRAG-R, STATIC-99, VRS:SO). Main outcome measures are self- and externally-reported DRF changes well as offending behaviour characteristics.

**Results:**

By September 20, 2023, N=12 individuals were enrolled in the treatment program. All individuals had a deviant sexual preference (exclusive/non-exclusive pedo-/hebephilia). Nine individuals reported past and/or current use of CSAI. Of these, two individuals reported at least one CSA in the past. Three had no previous use of CSAI or CSA history.

In the first treatment group (N=6), preliminary results show reduction in dynamic risk factors (e.g., Cognitive Bias, Sexual Compulsivity, Impulsivity) after the first 12 weeks of treatment. The evaluation of additional clinical data is pending.

**Conclusions:**

To date, therapy for individuals with pedophilia or hebephilia has been insufficient – particularly when not offending. Ongoing evaluation of the therapy program should provide further insight into responsiveness and therapeutic motivation of this target group. In particular, the impact of therapy on changing dynamic risk factors for CSA and CSAI remains to be examined.

**Disclosure of Interest:**

None Declared

